# Structure and dynamics of the gut bacterial microbiota of the bark beetle, *Dendroctonus rhizophagus* (Curculionidae: Scolytinae) across their life stages

**DOI:** 10.1371/journal.pone.0175470

**Published:** 2017-04-13

**Authors:** Carlos Iván Briones-Roblero, Juan Alfredo Hernández-García, Roman Gonzalez-Escobedo, L. Viridiana Soto-Robles, Flor N. Rivera-Orduña, Gerardo Zúñiga

**Affiliations:** 1 Posgrado en Ciencias Quimicobiológicas, Instituto Politécnico Nacional, Ciudad de México, México; 2 Departamento de Zoología, Instituto Politécnico Nacional, Ciudad de México, México; 3 Departamento de Microbiología, Instituto Politécnico Nacional, Ciudad de México, México; University of Illinois at Urbana-Champaign, UNITED STATES

## Abstract

Bark beetles play an important role as agents of natural renovation and regeneration in coniferous forests. Several studies have documented the metabolic capacity of bacteria associated with the gut, body surface, and oral secretions of these insects; however, little is known about how the bacterial community structure changes during the life cycle of the beetles. This study represents the first comprehensive analysis of the bacterial community of the gut of the bark beetle *D*. *rhizophagus* during the insect’s life cycle using 454 pyrosequencing. A total of 4 bacterial phyla, 7 classes, 15 families and 23 genera were identified. The *α*-diversity was low, as demonstrated in previous studies. The dominant bacterial taxa belonged to the Enterobacteriaceae and Pseudomonadaceae families. This low *α*-diversity can be attributed to the presence of defensive chemical compounds in conifers or due to different morpho-physiological factors in the gut of these insects acting as strong selective factors. Members of the genera *Rahnella*, *Serratia*, *Pseudomonas* and *Propionibacterium* were found at all life stages, and the first three genera, particularly *Rahnella*, were predominant suggesting the presence of a core microbiome in the gut. Significant differences in *β*-diversity were observed, mainly due to bacterial taxa present at low frequencies and only in certain life stages. The predictive functional profiling indicated metabolic pathways related to metabolism of amino acids and carbohydrates, and membrane transport as the most significant in the community. These differences in the community structure might be due to several selective factors, such as gut compartmentalization, physicochemical conditions, and microbial interactions.

## Introduction

The ecological opportunities and evolutionary history of species have shaped ecological interactions between species over long periods of time [1, 2]. The diversity of bark beetles (Curculionidae: Scolytinae), as reported for many other phytophagous beetle groups, is correlated with the diversification of their host [3, 4]. In the case of bark beetles, this interaction has determined their diversity of life history strategies (ecological, demographical or reproductive) as well as the development of morphological, physiological and behavioural adaptations due to their feeding habits on dead, stressed or healthy trees [5]. Their association with microorganisms (e.g., fungi and bacteria) has been a crucial and favorable relationship because it has enhanced their capacity for colonization, adaptation to novel habitats, and exploitation of different tissues or parts of plants, thereby their diversification [6].

*Dendroctonus* bark beetles play an important role in coniferous forests as restorers and agents of natural regeneration; however, some species frequently exhibit population fluctuations that make them one of the most destructive agents of these forests [7]. The colonization of healthy trees is not an easy task for adult beetles because they must bore towards the phloem against a dynamic and elaborated defence system composed of the bark thickness, the presence of phenolic compounds and terpenoids such as monoterpenes, diterpenes and sesquiterpenes, constitutive and traumatic resin ducts, and specialized phloem parenchyma cells [8]. Once inside of the tree, beetles copulate and excavate galleries where females oviposit. Then, larvae feed and develop on the phloem where they enlarge galleries that terminate in pupal chambers, from which brood adults finally emerge [9].

A number of studies have shown that filamentous fungi and yeasts associated with *Dendroctonus* bark beetles play important roles in colonization, nutrition, xenobiotic detoxification and pheromone production [10–14]. In the case of bacteria, these organisms can contribute to the degradation of starch, lipids and esters [15] as well as complex polymers such as cellulose and xylan [15–17]. Bacteria also fix nitrogen [18, 19], recycle nitrogenous metabolic products [19], tolerate and degrade monoterpenes [14, 20, 21], mediate the growth and sporulation of associated fungi [22] and produce antibiotics that inhibit the growth of antagonistic fungi [23– 24].

Although these studies have demonstrated the enormous metabolic capacity of a small fraction of the bacterial community associated to bark beetles, little is known about the bacterial community structure (composition and abundance) and dynamics throughout the life cycle of these beetles. The majority of diversity studies conducted in *Dendroctonus* species have analyzed certain life stages [16, 18, 25–27], with exception of Hu et al. [28], who systematically analyzed three life stages in *D*. *armandi*. However, all these studies were based on culture-dependent or culture-independent techniques such as molecular cloning and denaturing gradient gel electrophoresis (DGGE), that possess a limited statistical coverage for the sample size (sequences number or size), which can lead to underestimates and biases in both the description and estimation of α and ß diversity (i.e. the number of species that comprise a single community and how they vary over time or space). In addition, it is also difficult to determine how species are functionally connected and how they change because community structures can vary widely between species, individuals, development stages, host physiological conditions and geographical locations.

*Dendroctonus rhizophagus* Thomas and Bright is an aggressive bark beetle endemic to Sierra Madre Occidental in Mexico where it attacks and kills seedlings and saplings (< 3 m tall, 10 cm diam.) of 11 pine species [29]. The entire life cycle from egg to adult is completed within a year and it is synchronous (i.e. the same developmental stage occurs at the same time throughout its distribution range). One or two pairs colonize individual trees, where they copulate and excavate a communal gallery in which female oviposits. Larvae are gregarious and complete their development feeding on phloem; later, they migrate towards the tree roots, where brood adults emerge [30].

A previous study on the gut bacterial community of this beetle using culturing, 16S rRNA sequencing and DGGE methods revealed a low total diversity and shifts in the composition at the genera level among 5^th^ instar larvae, pupae and adults [16]. However, to obtain a broader and more complete knowledge of the *α* and *ß* diversity as well as the community structure, it is possible to use approaches based on high-throughput sequencing, which have a better statistical power than conventional methods; independently of their potential systematic biases inherent to samples processing, amplification and pyrosequencing, that may alter read abundance and reduce the utility of diversity metrics [31].

In bark beetles, these approaches have been used to characterize bacterial and fungal symbionts in field larvae and adults and lab-rearing insects of *Dendroctonus micans*, *D*. *punctatus* and *D*. *valens* [32]; to characterize and compare the bacterial microbiome associated with the surface of cuticle, interior of the body, and galleries from the eastern larch beetle, *D*. *simplex* [33]; to evaluate whether the exclusivity of the bacterial community in host trees is attributable to the red turpentine beetle, *D*. *valens* [34]; and to characterize the bacterial community metagenome associated with the mountain pine beetle, *D*. *ponderosae*, as well as to identify bacterial genes putatively involved in terpene degradation [35].

Given that phloem consumption is different among distinct developmental stages in *Dendroctonus* species and the nutritional quality of this substrate vary after the initial colonization of the host, we analyzed the *α* and *ß* diversity of the bacterial community in the gut of *D*. *rhizophagus* to determine: a) the change on the bacterial community structure across the insect life cycle, b) the exclusivity degree of community in different life stages, c) the persistence of the dominant taxa and, d) the predictive functional profile of the community using 16S rRNA sequences.

## Materials and methods

### Insect collection, dissection and DNA extraction

Larvae (5^th^ instar), pupae, teneral, pre-emerged adults and emerged adults were collected from naturally infested Arizona pines (60 cm high/ 10 cm diam., *Pinus arizonica* Engelm) in the lands of *Compañía Papelera Mexicana* (COPAMEX), in San Juanito locality, Bocoyna Municipality of Chihuahua, Mexico (27° 45′ 11″ N 107° 38′ 06″ W) in 2013. To conduct this part of the study, we got permission from the owners of COPAMEX. Because the life cycle of this species is annual and synchronous; larvae were collected in early-March, pupae in mid-April, teneral in mid-May, pre-emerged adults in mid-June, and emerged adults in mid-July.

Each developmental stage, except emerged adults, was collected from five different trees, which were extracted from the ground using pick and shovel. The bark of the roots was carefully removed with a knife and insects were taken with fine forceps. Emerged beetles were carefully collected directly from galleries built under the stem bark (5–10 cm below the ground level) of at least 20 newly colonized trees, because only one or two couples attack and kill a tree. All developmental stages were collected twice, from two different sets of trees and on the same dates, in order to integrate two biological replicates. All insects were placed in sterile plastic containers, stored at 4°C during their transport, and processed immediately once arrived to the laboratory.

Both biological replicates for each developmental stage, each composed by 30 specimens, were processed independently as described below. To remove body surface contaminants, specimens were sequentially rinsed with sterile distilled water for 1 min, a detergent solution (10 mM Tris-HCl pH 8, 1 mM EDTA, 10 mM NaCl; 1%SDS; 2% Triton X-100) for 1 min, a 1% sodium hypochlorite solution for 1 min, and a 70% ethanol solution for 1 min; finally, the specimens were repeatedly washed with sterile distilled water. To assess the efficiency of disinfection, the last washing water was inoculated in Petri dishes containing trypticase soya agar (TSA; BD Difco, US). Plates were incubated at 28°C for 48–72 h, no contamination was observed on the plates.

Insects were dissected in a drop of phosphate-buffered solution (PBS, pH 7.4; 137 mM NaCl, 2.7 mM KCl, 10 mM NaHPO_4_, 2 mM KH_2_PO_4_) under aseptic conditions in a laminar flow hood using fine-tipped forceps. To remove the gut, a longitudinal incision was made on the body of each of the immature stages; in the case of pre-emerged and emerged adults, elytra, wings, and tergites were removed before the longitudinal incision. In both biological replicates, for each development stage, three sets of ten guts were pooled to integrate three technical replicates. Each independently was placed in a 1.5-mL microfuge tube containing 1000 μL of PBS. Each set of ten guts was macerated using a sterile plastic pestle, and 200 μl were used for total genomic DNA extraction with a DNeasy Blood and Tissue kit (Qiagen, Valencia, CA) according to the manufacturer protocol for Gram-positive bacteria. DNA concentrations were quantified using a NanoDrop 2000cspectrophotometer(Thermo Scientific, Wilmington, DE).

### Bacterial 16S rRNA PCR amplification and pyrosequencing

Total genomic DNA from each set of ten guts, for developmental stage, was amplified separately with 8F and 556R primers [36], which were tagged with 10 bp multiplex identifiers (MID) and a Roche 454 adaptor for the Lib-L protocol. The V1-V3 region of the prokaryotic small-subunit (16S) rRNA gene was amplified. PCR amplification was performed in a final volume of 25 μL using a TC-142 5000 thermocycler (Techne, Stanffordshire, UK). Each reaction contained 80 ng of DNA template, 1x reaction buffer, 2.0 mM MgCl2, 0.4 pM each primer, 0.4 mM each dNTPs, and 1.0 U of Platinum Taq DNA polymerase High Fidelity (Invitrogen^™^ Life Sciences, US). The PCR conditions were as follow: initial denaturation at 94°C for 5 min; 25 cycles of denaturation at 94°C for 50 s, annealing at 53°C for 50 s and extension at 72°C for 50 s; and a final extension 72°C for 5 min. The triplicates of each sample were pooled prior to purification using a QIAquick Gel Extraction kit (Qiagen, Valencia, CA) according to the manufacturer’s protocol and quantified using a Nanodrop 2000c spectrophotometer (Thermo Scientific, Wilmington, DE). Amplicons obtained from the three PCR reactions of each developmental stage were pooled in equimolar concentrations of 50 μg for pyrosequencing using a Roche GS-FLX Titanium 454 pyrosequencer (Roche, Mannheim, DE) at Macrogen Inc. (Seoul, KR).

### Data processing and analysis

Data analysis was conducted using the software Quantitative Insight into Microbial Ecology (QIIME) version 1.8 [37]. All low-quality reads (Phred quality score < 25) and sequences <200 or > 500 bp long, containing ambiguous characters, homopolymers >6 bp and mismatches in primers > 14 were removed from subsequent analyses.

The high-quality sequences were sorted into operational taxonomic units (OTUs) according to the open-reference method at a 97% of similarity threshold using Uclust algorithm [38]. Chimeric sequences were detected and eliminated using Chimera Slayer [39]. All representative sequences for each OTU were aligned using PyNast [37] and the taxonomic assignment to different levels, from phylum to genus, was performed at an 80% confidence threshold using the Ribosomal Database Project (RDP) naïve Bayesian Classifier (http://rdp.cme.msu.edu/classifier/classifier.jsp) [40]. For taxonomic assignment of unclassified OTUs, representative sequences were manually corroborated against reference sequences deposited in the RDP (http://rdp.cme.msu.edu/seqmatch/seqmatch_intro.jsp), GenBank (http://blast.ncbi.nlm.nih.gov/Blast.cgi) databases. Reference sequences with the closest match were downloaded from the GenBank database, aligned with query sequences, and trimmed at the 5’ and 3’ ends. A phylogenetic inference analysis was performed using the maximum likelihood algorithm in PhyML (http://www.atgc-montpellier.fr/phyml/); *Anabaena variabilis* (NR_074300) was included as an outgroup.

Different estimators of bacterial species richness (Chao1) and *α*-diversity (Simpson's Reciprocal Index and Phylogenetic Diversity) were calculated [41–42] for each developmental stage and replicates. To estimate these indices, the number of reads was previously homogenized with respect to the sample with the lowest read counts. Due to the variances were heterogeneous, differences in the means of richness and diversity values were tested using Welch test. To determine the probability that a randomly selected amplicon from a sample was previously sequenced, the Good’s coverage was calculated as an estimator of sampling completeness [43].

The *β*-diversity of gut bacterial communities was estimated using unweighted and weighted Fast UniFrac distances. Weighted UniFrac analysis is based on the phylogenetic richness and the relative abundance of OTUs in each developmental stage, whereas unweighted UniFrac only considers richness [44]. This information is extracted from a phylogenetic tree constructed using the maximum likelihood method. The tree was computed in Fast Tree [45] using the generalized time reversible (GTR) nucleotide model. A Monte Carlo test was used to determinate whether *β*-diversity values among bacterial communities were statistically significant after 1000 randomizations of the original matrices. Finally, a principal coordinates analysis (PCoA) using both UniFrac distances was conducted to compare communities across life stages and replicates.

### Metagenome predictions from 16S rRNA surveys and functional analysis

We used PICRUSt (Phylogenetic Investigation of Communities by Reconstruction of Unobserved States) [46] to predict functional profile of bacterial communities indifferent life stages. An OTU table following the closed-reference picked method with QIIME was generated, after that sequences were demultiplexed. This table, in biom-format, was normalized and then used in PICRUSt to explore the metagenomic functional predictions. To evaluate the accuracy of predictions in the samples, we calculated The Nearest Sequenced Taxon Index (NSTI). The NSTI scores summarize the extent to which microorganism in a sample are related to sequences genomes, and they represent the average branch length that separates each OTU in a sample from a reference bacterial genome, weighting their relative abundance in the each sample. Low values of this index indicate a closer mean relationship. The table containing the predicted gene family-counts per sample, based on orthologous groups and identifiers following Kyoto Encyclopedia of Genes and Genomes (KEGG)(www.genome.jp/kegg/) at level 2, were cleaned following the criteria: removal of categories unrelated with bacterial physiology/metabolism (like human diseases), and removal of gene family categories with count equal to 0. The database was finally used to generate a heatmap in CIMminer (https://discover.nci.nih.gov/cimminer).

## Results

A set of 203,012 sequences was obtained from the nine samples submitted for pyrosequencing. The pupae II replicate was eliminated because the length of the sequences was < 200 bp. After applying the quality control standards, a total of 120,181 sequences with an average length of 460 nts remained for the statistical analysis and a total of 310 OTUs were defined at 97% of sequence similarity. The final number of obtained reads ranged from 7600 to 26,400 sequences per sample; thus, all samples were homogenized to 7600 reads.

### Abundance of bacteria associated with the gut of *D*. *rhizophagus*

**Fig 1:**
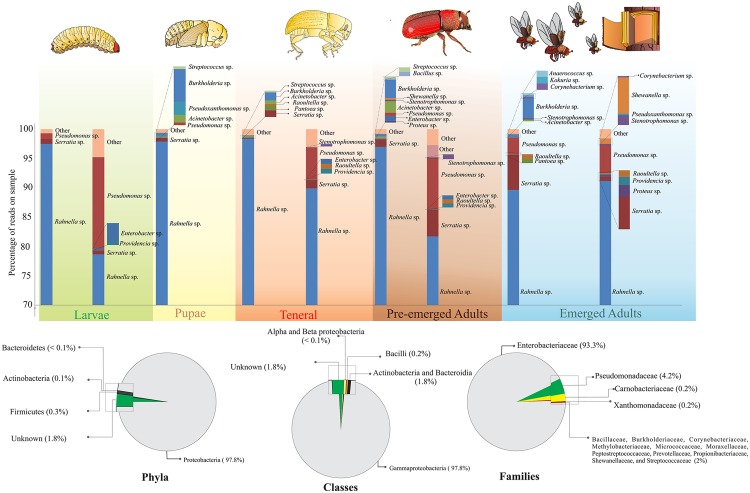
Relative abundance of bacterial taxa at phylum, class, family and genus levels in the gut of different life stages of *Dendroctonus rhizophagus*. High-quality sequences were sorted into operational taxonomic units (OTUs) according to the open-reference method at a 97% of similarity.

A total of 4 bacterial phyla, 7 classes, 15 families, and 23 genera were identified across all life stages (Fig 1). Proteobacteria (97.8%) was the most abundant phylum, followed by Firmicutes (0.3%), Actinobacteria (0.1%), and Bacteroidetes (< 0.1%) (Fig 1). At the genus level, *Rahnella* (91%) was the best represented, followed by *Pseudomonas* (4.2%), *Serratia* (1.8%), and other genera as *Enterobacter*, *Pantoea*, *Proteus*, *Providencia*, *Raoultella*, *Carnobacterium*, *Stenotrophomonas*, *Propionibacterium*, *Acinetobacter*, *Anaerococcus*, *Bacillus*, *Burkholderia*, *Corynebacterium*, *Kocuria*, *Lactococcus*, *Methylobacterium*, *Prevotella*, *Pseudoxanthomonas*, *Shewanella* and *Streptococcus*, that together corresponded to <2.0% (Fig 1 and S1 Fig). Only 1.8% of total reads were unassignable (Fig 1).

### Diversity and developmental stages

The Good’s coverage was greater than 99.4% in all life stage samples, indicating adequate sampling completeness (Table 1). The expected richness metric (Chao 1) was significantly different among life stages (Welch *F* test: F = 25.63, *p* < 0.05); the highest richness was observed in emerged and pre-emerged adults, while the lowest value was found in the larval stage (Table 1). The Simpson´s reciprocal index, which indicates the number of dominant OTUs, was not statistically significant among all developmental stages indicating that only one OTU was predominant in them (Table 1). The PD index showed statistical differences, indicating a higher diversity in pre-emerged and emerged adults (ANOVA Welch *F* test: F = 5.32, *p* < 0.05) (Table 1).

**Table 1:** Richness and alpha diversity estimation of the bacterial community in the gut of different life stages of *D*. *rhizophagus*.

Developmental stage	OTUs observed^a^	Good'scoverage (%)^a^	Chao1^a^	Simpson's reciprocal (1/D)^a^	PD^a^^,^^b^
Larvae	55	99.6	72	1.3	0.80
Pupae	40	99.7	78	1.4	1.45
Teneral	65	99.5	116	1.2	1.11
Pre-emerged adults	73	99.5	122	1.4	1.9
Emerged adults	64	99.6	122	1.4	1.7

^a^ Mean values between two biological replicates for each life stage, except in pupae.

^b^ Phylogenetic diversity index

The genera *Rahnella*, *Pseudomonas*, *Serratia*, and *Propionibacterium* were persistent in all life stages and replicates. The genera *Acinetobacter*, *Enterobacter*, *Providencia*, *Raoultella*, and *Stenotrophomonas* were identified in almost all life stages, but not consistently found in both replicates. Finally, *Anaerococcus*, *Carnobacterium*, *Corynebacterium*, *Bacillus*, *Burkholderia*, *Kocuria*, *Lactococcus*, *Methylobacterium*, *Prevotella*, *Proteus*, *Pseudoxanthomonas*, and *Shewanella* were only found in one development stage or replicate, with the exception of *Pantoea* and *Streptococcus*, which were recovered in three life stages (S1 Table).

**Fig 2:**
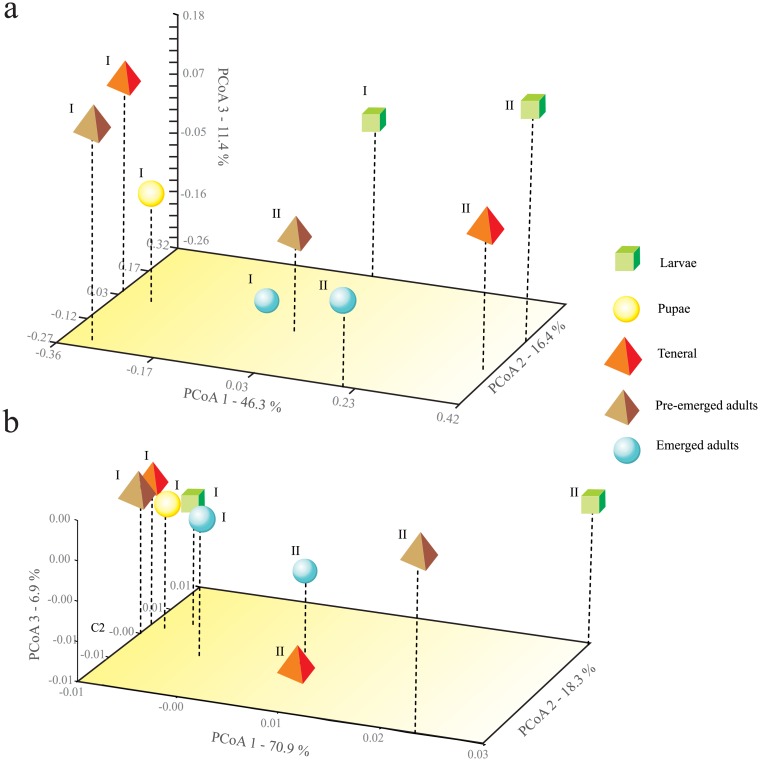
Principal Coordinates Analysis (PCoA) of the bacterial community in the gut of different life stages of *D*. *rhizophagus*. The PCoA was conducted using unweighted (a) and weighted (b) pairwise UniFrac distances among communities of life stages and replicates.

The first three principal coordinates of the PCoA, both with weighted and unweighted UniFrac distances, explained 96.3% and 74.1% of the total observed variation, respectively (Fig 2). The PCoA using weighted UniFrac distance showed no significant differences among the *β*-diversity of the bacterial communities of the different life stages and replicates (Fig 2b); in contrast, the PCoA using unweighted UniFrac showed statistically significant differences (*P*<0.5) (Fig 2a).

### Functional profiling prediction

**Fig 3:**
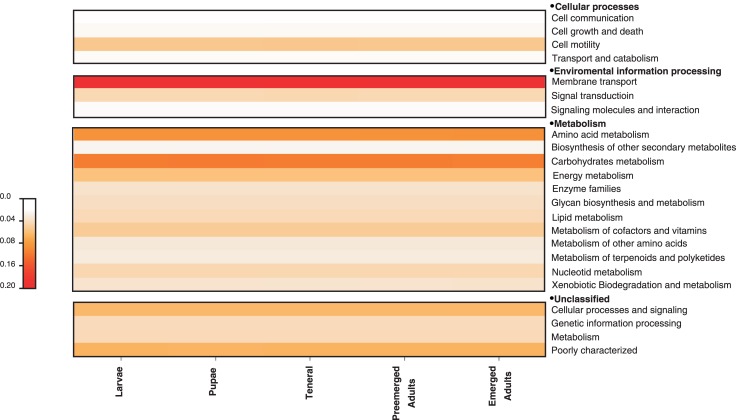
Heatmap of relative abundances of the most important metabolic pathways inferred by PICRUSt in the gut bacterial community of *D*. *rhizophagus* across its life cycle. Warm colors represent high abundances and clear colours represent low abundances.

The NSTI index values varied from 0.025 (larvae) to 0.028 (teneral), with a mean of 0.026 ± 0.001. This range indicates that bacterial functional gene predictions were accurate in all the libraries. Among the predicted KEGG pathways, the 3 most significant were those related to metabolism of amino acids and carbohydrates, and membrane transport, representing around 41.84% of total pathways. Other important pathways found in a lower proportion were: metabolism of terpenoids, poliketydes, lipids, and xenobiotics (Fig 3).

## Discussion

This study represents the first comprehensive analysis of the bacterial community in the gut of the bark beetle *D*. *rhizophagus* throughout its life cycle using 454 pyrosequencing. A total of 4 phyla, 7 classes, 15 families, and 23 genera comprised the bacterial community in all life stages; at the genus level, only *Rahnella*, *Serratia*, *Pseudomonas*, and *Propionibacterium* were recorded in all development stages, being *Rahnella* the most abundant.

Previous *α*-diversitystudies reported from 5 to 11 bacterial genera in specific life stages of some species of the *Dendroctonus* and *Ips* genera using culture-dependent and culture-independent methods [16, 18, 25–28, 47–49]. In the particular case of *D*. *rhizophagus*, 7 (*Acinetobacter*, *Kocuria*, *Pseudomonas*, *Propionibacterium*, *Rahnella*, *Raoultella*, and *Stenotrophomonas*) of the 9 bacterial genera previously recovered in the gut of larvae, pupae, and adults using culturing, DGGE, and 16S rRNA libraries [16] were also detected in the present survey. This is equivalent to 30% of the total bacterial genera recovered in this study using pyrosequencing. These differences reflect the limited scope of conventional methods, which severely underestimate the microbial community diversity; however, the culture methods are complementary and necessary to explore functional capacities of members of the bacterial community.

The low *α*-diversity observed in this and other *Dendroctonus* species using conventional techniques and 16S rRNA gene pyrosequencing [32–34], confirm that these bark beetles harbor bacterial communities that generally present lower richness and phylogenetic diversity than other phytophagous insects whose communities has also been characterized using both approaches [50–53]. Whereas several authors have argued that the low *α*-diversity observed in bark beetles can be attributed to the presence of defensive chemical compounds in conifers [20], an alternative hypothesis is to assume that different morpho-physiological factors in the gut of these insects act as strong selective factors structuring the microbial communities in these bark beetles.

In fact, the comparison of endophytic bacterial communities (bark, roots and phloem) of Arizona pine (*Pinus arizonica*) saplings (one of the preferred pine host by *D*. *rhizophagus* [29]) and bacterial community found in this study, shows that the most abundant members in the gut (*Rahnella*, *Serratia*, *Pseudomonas*, and *Propionibacterium*) of this bark beetle are present also in tissues of non-colonized trees (S2 Fig), suggesting that some microorganisms are acquired from the phloem through feeding (pers. comm). So, the ideas that defensive chemical compounds in conifers or morpho-physiological factor can select the presence of bacteria in the gut should to be borne out.

Our results also demonstrate that the *β*-diversity change during the insect's life cycle. Similar results have been reported in other *Dendroctonus* species using culture methods, DGGE, and molecular cloning [16, 25, 28, 47]. Unfortunately, it has been difficult to determine whether these differences are consistent, because these last studies did not systematically analyze the community across different developmental stages, with exception of *D*. *armandi*, where differences in the community structure were observed through several life stages [28].

The PCoA analysis using unweighted UniFrac distances indicates that *β*-diversity is statistically significant among bacterial communities in the different life stages and replicates; however, the same analysis using weighted UniFrac distances did not show significant differences. These differences are given by those low-abundant bacteria (< 0.1% reads) present in some post-larval stages (e.g., *Bacillus* sp., *Burkholderia* sp., *Kocuria* sp., *Prevotella* sp., *Pseudoxanthomonas* sp., *Streptococcus* sp., and *Shewanella* sp.). These changes in the community structure are also observed between replicates, indicating that bacterial community may differ also between conspecifics. Differences in the *β*-diversity have also been observed in the analysis of the endomicrobiome and ectomicrobiome of the bark beetle *D*. *simplex* by the presence or absence of low-abundance taxa [33]. However, due to the low number of replicates in this study, some caution in interpreting inferences about the presence or absence of particular bacterial genera in the gut of the different life stages is prudent. The error associated with the molecular technique used should not be discarded.

Although there is not a reliable explanation why the presence and relative abundance of the non-dominant bacteria vary among different life stages and replicates, we hypothesize that dominant bacteria (*Rahnella*, *Serratia*, and *Pseudomonas*), along the insect's immune system, may regulate the gut bacterial community. It has been demonstrated that these bacteria possess antimicrobial activity and regulation mechanisms of bacterial populations (quorum sensing) [54–57]. In addition, experimental evidence indicate that the response of the innate immune system of *Dendroctonus* species vary across of their different developmental stages, being higher in immature stages [58].

Based on the detection of strictly anaerobic bacteria (i.e., *Prevotella* in this study and Clostridiales in other *Dendroctonus* species) [33], facultative anaerobes and strict aerobes in this study, we argue that other factors that may compromise the population density of certain bacteriaarethe compartmentalization of the gut [59, 60], its histolysis in the larval stage, the histogenesis in new adults, and the existence of a physicochemically heterogeneous environment (i.e., pH, protease and lysozyme production, O_2_ gradients, redox potential), as demonstrated in other insect species [61–63].

The continuous presence of *Rahnella*, *Serratia*, *Providencia*, *Raoultella*, *Pseudomonas*, *Acinetobacter*, *Propionibacterium*, and *Strenotrophomonas* in all or at least four developmental stages, suggests the existence of a microbiome, i.e., a characteristic microbial community occupying a well-defined habitat in the gut of *D*. *rhizophagus*. Some metabolic capacities of bacteria belonging to this microbiome are known. For example, *Rahnella*, the dominant bacteria in this study, have been isolated from several *Dendroctonus* species [16, 25, 26]. Its abundance suggests an important dietary contribution as well roles in the detoxification processes for *D*. *rhizhopagus*. In fact, these bacteria recovered from *D*. *valens* and *D*. *rhizophagus* are able to recycle uric acid, which could increase the availability of nitrogen to bark beetles [18]. In addition, it has been shown that this bacterium show esterase activity in *D*. *rhizophagus* [15], which might be associated with the degradation of glycerol esters, short-chain triglycerides, and partially ester bonds present in xylan, aromatic hydrocarbons, insecticides, aromatic esters, phenolic acids and resin acids [64].

Likewise, bacteria in the genera *Pseudomonas*, *Serratia*, *Stenotrophomonas*, *Kocuria*, *Methylobacterium*, and *Pseudoxanthomonas* isolated from *D*. *rhizophagus* and *D*. *armandi* are able to degrade cellulose [15–17]. *Acinetobacter* and *Pseudomonas* isolated from *D*. *rhizophagus* have also been shown to be implicated on lipolytic and esterase activities, and *Pseudomonas* has been shown to have amylolytic and xylanolytic activities [15], which may complement the nutritional capacity of these bark beetles by allowing the use of phloem as a food source. Finally, the genus *Pseudomonas*, isolated from *D*. *terebrans* [65], and *Raoultella*, from *D*. *rhizophagus* and *D*. *valens*, have shown diazotrophic activity in vitro [18], which could contribute to the nitrogen balance in insects.

Of particular interest is the presence of *Rahnella*, *Serratia*, *Pseudomonas* and *Propionibacterium* in all life stages, which suggests that they might represent a core within the microbiome. The persistence of these bacteria in the gut and the high relative abundance, particularly of *Rahnella*, *Serratia*, and *Pseudomonas*, suggest that these bacteria can maintain stable populations in this habit, thereby ensuring a set of physiological functions for the insect (*e*.*g*., polysaccharide and lipid degradation, terpenoid metabolism, nutrient allocation, nitrogen fixation, and nitrogen compound recycling) in various life stages. In fact, the predictive metabolic profiling in PICRUSt supports some of these metabolic capacities.

However, as also suggest our predictive analysis, the whole bacterial community, rather than dominant taxa, might be involved in these biochemical functions. If this idea is correct, we could expect core metabolic pathways where all members of the community participate. Whereas we have not experimental data (e.g., “meta-omics” approaches) to support it, the genome sequences of the dominant bacteria (*Rahnella*, *Serratia*, and *Pseudomonas*) isolated from *D*. *rhizophagus* reveal the presence of orthologous genes involved in the nitrogen metabolism (e.g., ammonium assimilation and nitrate ammonification) and carbohydrates metabolism (e.g., starch hydrolysis and glycogen synthesis)(pers. comm.) (S3 and S4 Figs).

Knowing how selective factors influence the community structure and dynamics is fundamental to understand how bacteria can persist in a heterogeneous and changing environment such as the gut of *D*. *rhizophagus*. In addition, this will allow us to understand how the bark beetles gut works and how the bacterial community to carry out a set of core metabolic pathways in for the benefit of its hosts and themselves.

### Sequence submission

The raw data obtained by 454-based pyrosequencing was submitted to the Short Read Archive database at NCBI (http://www.ncbi.nlm.nih.gov) (Accession SRP#: SRP066495).

## Supporting information

S1 Fig Maximum likelihood phylogenetic tree of only one representative sequence for each of 23 OTUs assigned to the genus level. The model GTR (-lnL = 6301.51, freqA = 0.2786, freqC = 0.2086, freqG = 0.3196, freqT = 0.1932) was used for the analysis. *Anabaena variabilis* (NR_074300) was used as outgroup. The confidence at each node was assessed by 1,000 pseudo-replicates and bootstrap support values are indicated for major nodes having values ≥ 50%. The scale bar indicates substitution/site.
(PDF)

S2 Fig Maximum likelihood phylogenetic tree of representative sequences of pyrosequenced bacterial OTUs of this study, endophytic bacterial OTUs of Arizona pine and GenBank database sequences. The model GTR+I+G (-lnL = 6441.55, I = 0.207, G = 0.752, freqA = 0.24322, freqC = 0.20534, freqG = 0.34238, freqT = 0.20906) was used for the analysis. *Anabaena variabilis* (NR_074300) was used as outgroup. The robustness at each node was assessed after 1,000 pseudo-replicates and bootstrap support values are indicated with grey circles for major nodes having values ≥ 50%. The scale bar indicates substitution/site.
(PDF)

S3 Fig Predicted protein putatively involved in metabolism of starch/glycogen of dominants members (*Rahnella*, *Pseudomonas* and *Serratia*) in the *Dendroctonus rhizophagus* gut. Proteins were identified in each genome of these bacteria using either Kyoto Encyclopedia of Genes and Genomes (KEGG), and BLASTp in GeneBank, Pfam, and UniProt. **Panel A.** Proposed pathway for metabolism of strach/glycogen. The semicircle represents the putative protein and the metabolic step where it participates, the geometric symbol the bacteria with these putative proteins. *glgA* (glycogen synthase), *glgE* (maltosyltransferase), *gbe* (glycogen branching enzyme), *glgX* (glycogen debranching enzyme), *malQ* (glucanotransferase), *amyCP* and *gla* (cytoplasmic amylase, periplasmic amylase and glucoamylase). Trehalose biosynthesis: *treY* (maltooligosyl trehalose synthase), *treS* (trehalose synthase), *treZ* (malto-oligosyltrehalose trehalohydrolase), *tps* (trehalose-phosphate synthase), and *treP* genes (trehalose phosphorylase). **Panel B**. A gene cluster involved in the utilization of maltose and maltodextrin only in *Serratia*. The cluster is integrated by maltose operon *malEFG*(maltose/maltodextrin ABC transporters), and other adjacent genes *malZ*, *malK*, *lamB* and *malM* (maltodextrin glucosidase, maltose/maltodextrin transport ATP-binding protein, maltoporin, and maltose operon periplasmic protein).
(PDF)

S4 Fig Predicted protein putatively involved in nitrogen metabolism and amino acids synthesis of dominants members (*Rahnella*, *Pseudomonas* and *Serratia*) in the *Dendroctonus rhizophagus* gut. Proteins were identified in each genome of these bacteria using either Kyoto Encyclopedia of Genes and Genomes (KEGG), and BLASTp in GeneBank. The semicircle represents the putative protein and the metabolic step where it participates, the geometric symbol the bacteria with these putative proteins; *Raoultella* is included based on experimental data (Morales-Jiménez et al. 2013). Assimilatory nitrate reduction process: *nar GHIJ* (respiratory nitrate reductase), and *niR* genes (nitrite reductase). Nitrogen fixation process: *nifD* (nitrogenase molybdenum-iron protein), and *nifH* genes (nitrogenase iron protein). Amino acids synthesis: *glnA* (glutamine synthase), *gls* (glutamate synthase-dependent ferredoxin), and *gltB genes* (NADPH-dependent glutamate synthase).
(PDF)

S1 Table Diversity and abundance of the bacterial community in the gut of *D*. *rhizophagus* across the different life stages. It is shown the number of reads for each taxa (genera level) from samples homogenized with respect to the sample with the lowest reads counts (7600 reads).
(DOCX)
