# Supplementary figures and images for: Structure and dynamics of the gut bacterial microbiota of the bark beetle, Dendroctonus rhizophagus (Curculionidae: Scolytinae) across their life stages
Source: PLoS One. 2017 Apr 13;12(4):e0175470. doi: 10.1371/journal.pone.0175470 (PMC5391025; doi:10.1371/journal.pone.0175470)

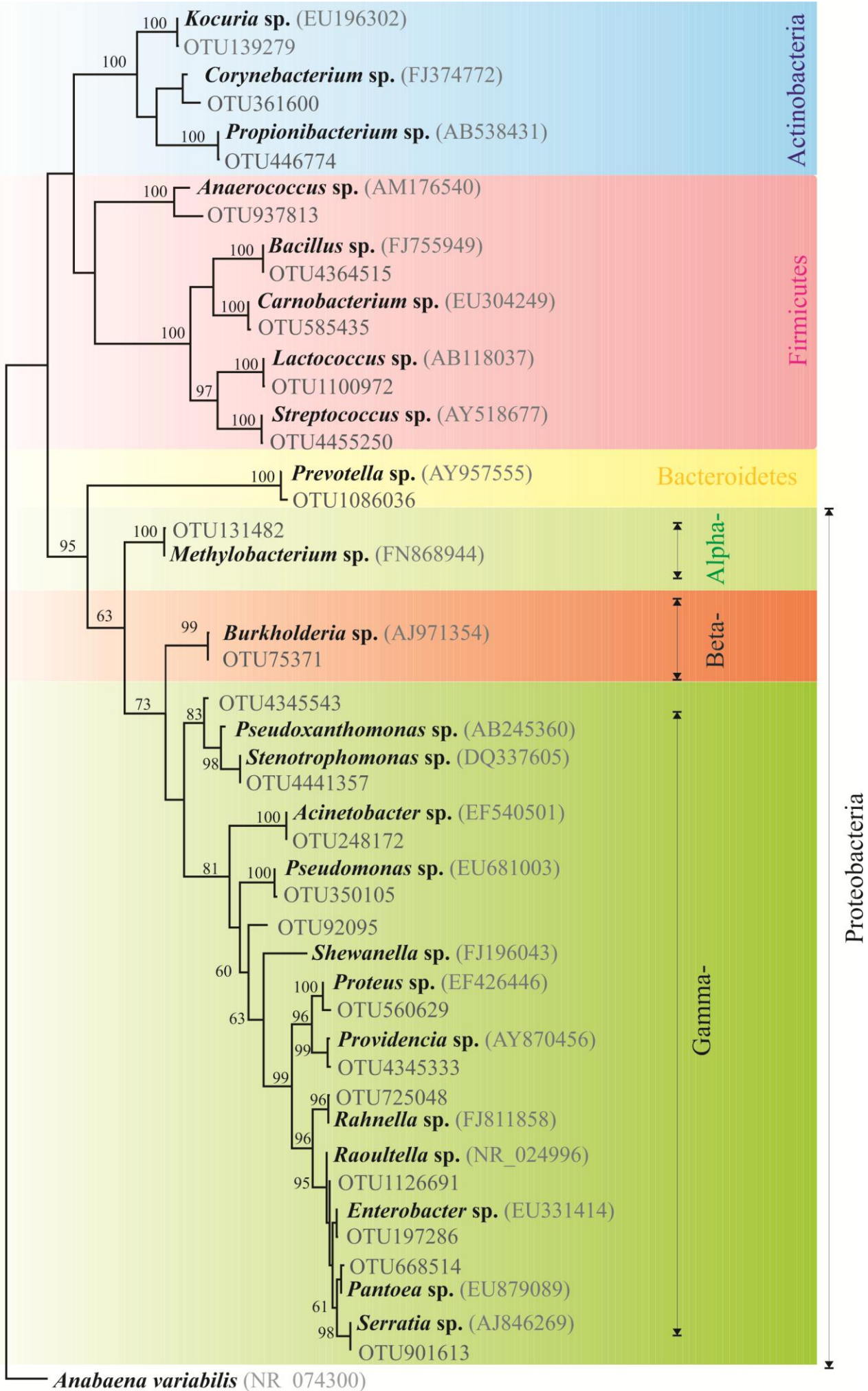

0.06

Supplement: S1 Fig — The model GTR (-lnL = 6301.51, freqA = 0.2786, freqC = 0.2086, freqG = 0.3196, freqT = 0.1932) was used for the analysis. Anabaena variabilis (NR_074300) was used as outgroup. The confidence at each node was assessed by 1,000 pseudo-replicates and bootstrap support values are indicated for major nodes having values ≥ 50%. The scale bar indicates substitution/site. (PDF) [file pone.0175470.s001.pdf]

Tree scale: 0.1

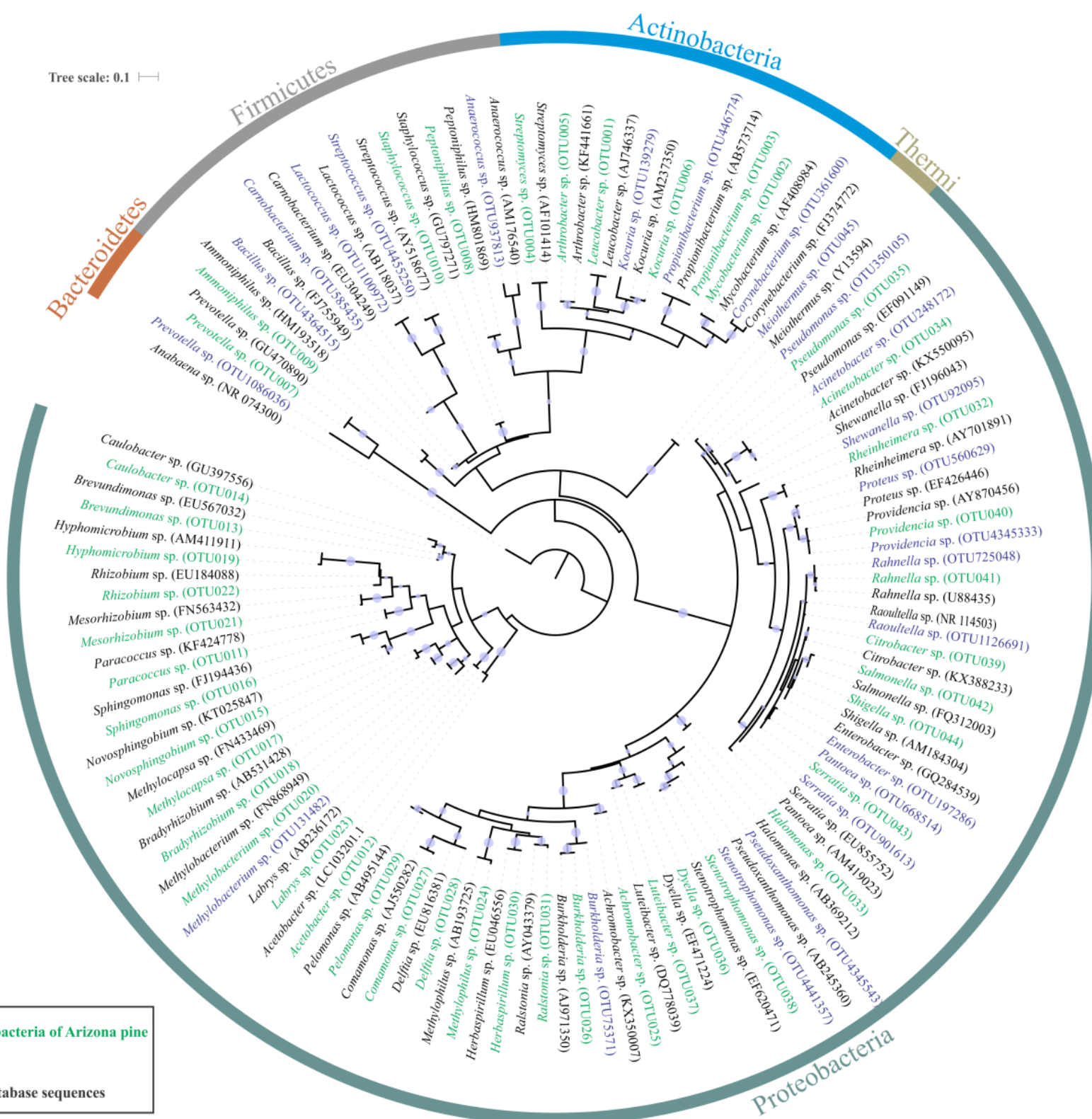

## Endophytic bacteria of Arizona pine

### This study

### GenBank database sequences

Supplement: S2 Fig — The model GTR+I+G (-lnL = 6441.55, I = 0.207, G = 0.752, freqA = 0.24322, freqC = 0.20534, freqG = 0.34238, freqT = 0.20906) was used for the analysis. Anabaena variabilis (NR_074300) was used as outgroup. The robustness at each node was assessed after 1,000 pseudo-replicates and bootstrap support values are indicated with grey circles for major nodes having values ≥ 50%. The scale bar indicates substitution/site. (PDF) [file pone.0175470.s002.pdf]

## Starch/glycogen metabolism

A.

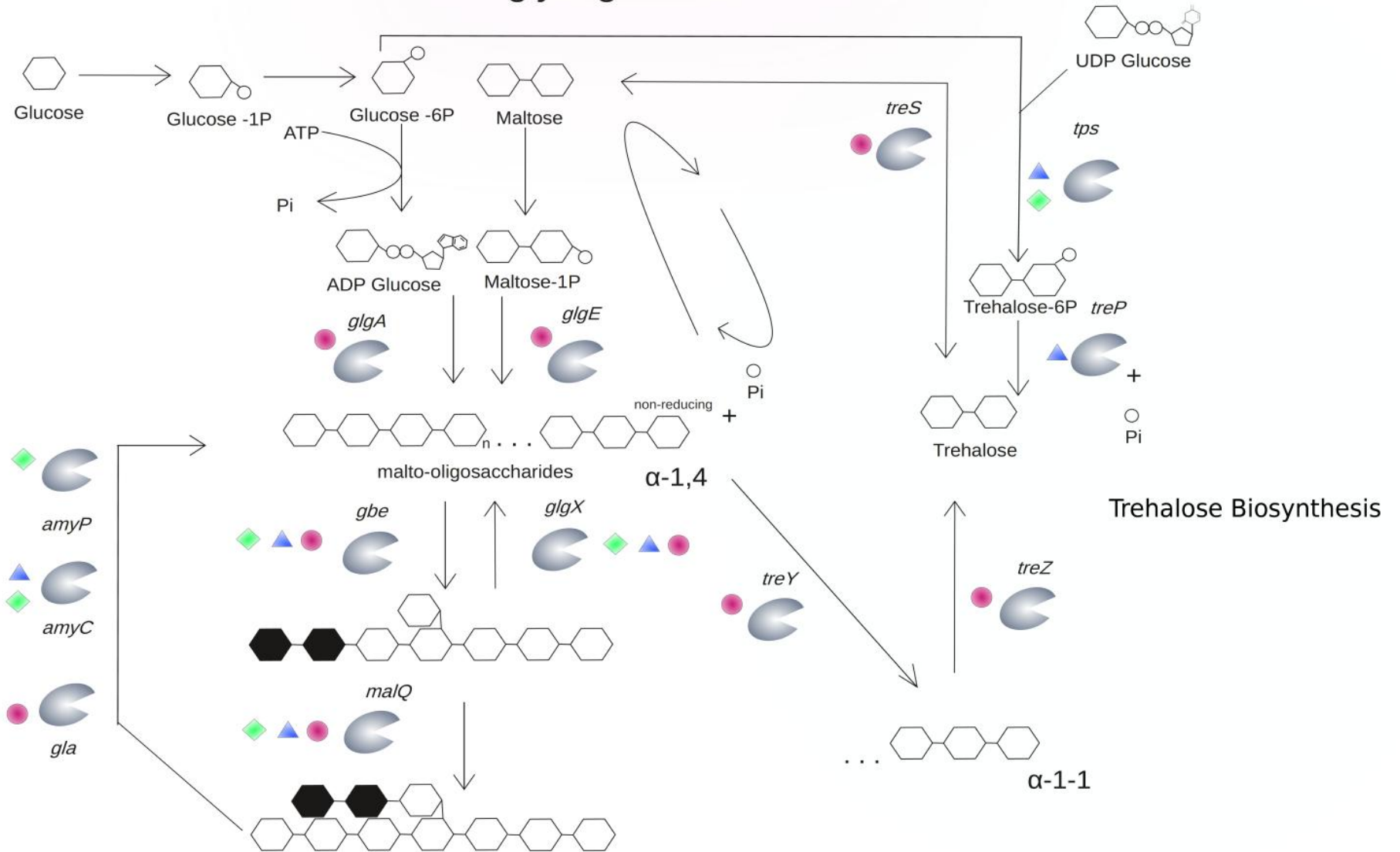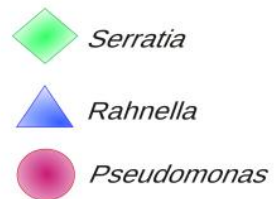

B.

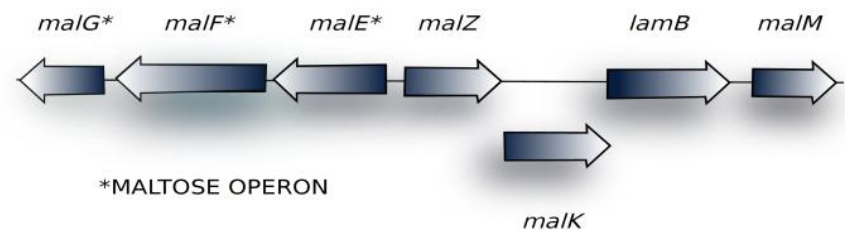

Supplement: S3 Fig — Proteins were identified in each genome of these bacteria using either Kyoto Encyclopedia of Genes and Genomes (KEGG), and BLASTp in GeneBank, Pfam, and UniProt. Panel A. Proposed pathway for metabolism of strach/glycogen. The semicircle represents the putative protein and the metabolic step where it participates, the geometric symbol the bacteria with these putative proteins. glgA (glycogen synthase), glgE (maltosyltransferase), gbe (glycogen branching enzyme), glgX (glycogen debranching enzyme), malQ (glucanotransferase), amyCP and gla (cytoplasmic amylase, periplasmic amylase and glucoamylase). Trehalose biosynthesis: treY (maltooligosyl trehalose synthase), treS (trehalose synthase), treZ (malto-oligosyltrehalose trehalohydrolase), tps (trehalose-phosphate synthase), and treP genes (trehalose phosphorylase). Panel B. A gene cluster involved in the utilization of maltose and maltodextrin only in Serratia. The cluster is integrated by maltose operon malEFG(maltose/maltodextrin ABC transporters), and other adjacent genes malZ, malK, lamB and malM (maltodextrin glucosidase, maltose/maltodextrin transport ATP-binding protein, maltoporin, and maltose operon periplasmic protein). (PDF) [file pone.0175470.s003.pdf]

# Nitrogen metabolism

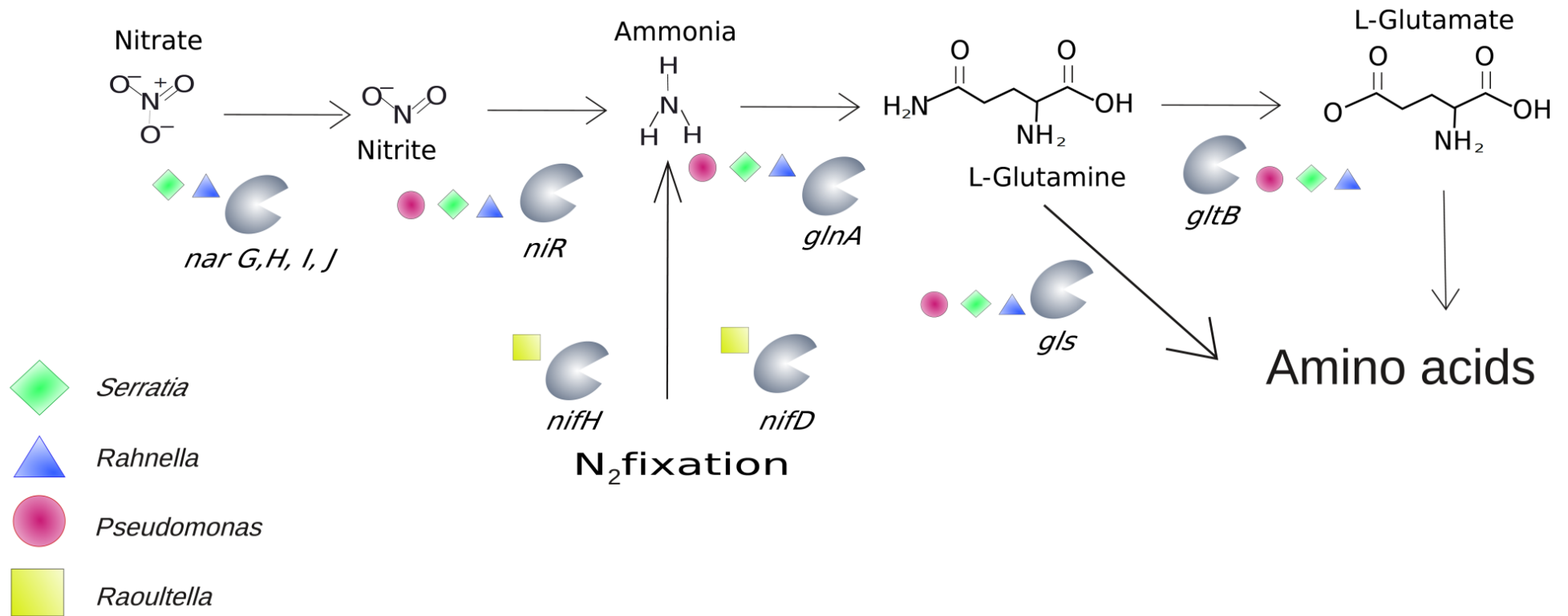

Supplement: S4 Fig — Proteins were identified in each genome of these bacteria using either Kyoto Encyclopedia of Genes and Genomes (KEGG), and BLASTp in GeneBank. The semicircle represents the putative protein and the metabolic step where it participates, the geometric symbol the bacteria with these putative proteins; Raoultella is included based on experimental data (Morales-Jiménez et al. 2013). Assimilatory nitrate reduction process: nar GHIJ (respiratory nitrate reductase), and niR genes (nitrite reductase). Nitrogen fixation process: nifD (nitrogenase molybdenum-iron protein), and nifH genes (nitrogenase iron protein). Amino acids synthesis: glnA (glutamine synthase), gls (glutamate synthase-dependent ferredoxin), and gltB genes (NADPH-dependent glutamate synthase). (PDF) [file pone.0175470.s004.pdf]
