# Supplementary material for: Structure and dynamics of the gut bacterial microbiota of the bark beetle, Dendroctonus rhizophagus (Curculionidae: Scolytinae) across their life stages
Source: PLoS One. 2017 Apr 13;12(4):e0175470. doi: 10.1371/journal.pone.0175470 (PMC5391025; doi:10.1371/journal.pone.0175470)
Supplement: S1 Table — It is shown the number of reads for each taxa (genera level) from samples homogenized with respect to the sample with the lowest reads counts (7600 reads). (DOCX) [file pone.0175470.s005.docx]

| **Table S1. Diversity and abundance of the bacterial community in the gut of *D. rhizophagus* across the different life stages.** It is shown the number of reads for each taxa (genera level) from samples homogenized with respect to the sample with the lowest reads counts (7600 reads). | | | | | | | | | | | | | | | | | |
| --- | --- | --- | --- | --- | --- | --- | --- | --- | --- | --- | --- | --- | --- | --- | --- | --- | --- |
|  | |  |  |  |  |  |  |  |  |  |  |  |  |  |  |  |  |
|  | |  |  |  |  |  | **Developmental stage** | | | | | | | |  |  |  |
| **Taxonomic Assignment** | | **Similariry**  **(%)** |  | Larvae I | Larvae  II |  | Pupae  I | |  | Teneral  I | Teneral  II |  | Pre-emerged  adults I | Pre-emerged  Adults II |  | Emerged  Adults I | Emerged  Adults II |
| **Proteobacteria** | |  |  |  |  |  |  | |  |  |  |  |  |  |  |  |  |
|  | **Gammaproteobacteria** |  |  |  |  |  |  | |  |  |  |  |  |  |  |  |  |
|  | **Enterobacteriaceae** |  |  |  |  |  |  | |  |  |  |  |  |  |  |  |  |
|  | *Rahnella* sp. ( FJ811858) | 99.78 |  | 7309 | 5914 |  | 7307 | |  | 7366 | 6585 |  | 7275 | 6070 |  | 6723 | 6867 |
|  | *Serratia* sp. ( AJ846269) | 100 |  | 69 | 49 |  | 46 | |  | 12 | 281 |  | 91 | 384 |  | 453 | 75 |
|  | *Pantoea* sp. (AM419023) | 97.48 |  | 0 | 0 |  | 0 | |  | 6 | 0 |  | 1 | 0 |  | 4 | 0 |
|  | *Proteus* sp. (EF426446) | 98.06 |  | 0 | 0 |  | 0 | |  | 0 | 0 |  | 1 | 0 |  | 0 | 18 |
|  | *Providencia* sp. (AY870456) | 98.51 |  | 0 | 4 |  | 0 | |  | 0 | 2 |  | 0 | 3 |  | 0 | 8 |
|  | *Raoultella* sp. (AF129442) | 99.74 |  | 0 | 0 |  | 1 | |  | 5 | 1 |  | 2 | 2 |  | 3 | 10 |
|  | *Enterobacter* sp.(GQ284539) | 99.76 |  | 0 | 2 |  | 0 | |  | 0 | 3 |  | 8 | 1 |  | 0 | 1 |
|  | **Pseudomonadaceae** |  |  |  |  |  |  | |  |  |  |  |  |  |  |  |  |
|  | *Pseudomonas* sp. ( EU681003) | 99.16 |  | 68 | 1172 |  | 3 | |  | 1 | 397 |  | 5 | 649 |  | 300 | 358 |
|  | **Moraxellaceae** |  |  |  |  |  |  | |  |  |  |  |  |  |  |  |  |
|  | *Acinetobacter* sp. (EF540501) | 99.79 |  | 0 | 0 |  | 5 | |  | 5 | 0 |  | 13 | 0 |  | 0 | 9 |
|  | **Xanthomonadaceae** |  |  |  |  |  |  | |  |  |  |  |  |  |  |  |  |
|  | *Stenotrophomonas* sp.  ( DQ337605) | 100 |  | 0 | 0 |  | 2 | |  | 1 | 6 |  | 3 | 34 |  | 4 | 48 |
|  | *Pseudoxaanthomonas* sp.  AB245360) | 98.34 |  | 0 | 0 |  | 14 | |  | 0 | 0 |  | 0 | 0 |  | 0 | 1 |
|  | **Shewanellaceae** |  |  |  |  |  |  | |  |  |  |  |  |  |  |  |  |
|  | *Shewanella* sp. (FJ196043) | 98.23 |  | 0 | 0 |  | 0 | |  | 0 | 0 |  | 1 | 0 |  | 0 | 21 |
|  | **Betaproteobacteria** |  |  |  |  |  |  | |  |  |  |  |  |  |  |  |  |
|  | **Burkholderiaceae** |  |  |  |  |  |  | |  |  |  |  |  |  |  |  |  |
|  | *Burkholderia* sp. (AJ971354) | 100 |  | 0 | 0 |  | 26 | |  | 0 | 0 |  | 0 | 0 |  | 0 | 0 |
|  | **Alphaproteobacteria** |  |  |  |  |  |  | |  |  |  |  |  |  |  |  |  |
|  | **Methylobacteriaceae** |  |  |  |  |  |  | |  |  |  |  |  |  |  |  |  |
|  | *Methylobacterium* sp. (FN868944) | 99.78 |  | 0 | 0 |  | 1 | |  | 0 | 0 |  | 0 | 0 |  | 0 | 0 |

| **Table S1 (continued). Diversity and abundance of the bacterial community in the gut of *D. rhizophagus* across the different life stages.** | | | | | | | | | | | | | | | | | | |
| --- | --- | --- | --- | --- | --- | --- | --- | --- | --- | --- | --- | --- | --- | --- | --- | --- | --- | --- |
|  | | |  |  |  |  |  |  |  |  |  |  |  |  |  |  |  |  |
|  | | |  |  |  |  |  | **Developmental stage** | | | | | | | |  |  |  |
| **Taxonomic Assignment** | | | **Similariry**  **(%)** |  | Larvae  I | Larvae  II |  | Pupae  I | |  | Teneral  I | Teneral  II |  | Pre-emerged  adults I | Pre-emerged  adults II |  | Emerged  Adults I | Emerged  Adults II |
| **Actinobacteria** | | |  |  |  |  |  |  | |  |  |  |  |  |  |  |  |  |
|  | **Actinobacteria** | |  |  |  |  |  |  | |  |  |  |  |  |  |  |  |  |
|  | **Propionibacteriaceae** | |  |  |  |  |  |  | |  |  |  |  |  |  |  |  |  |
|  | | *Propionibacterium* sp.  (AB538431) | 99.74 |  | 1 | 0 |  | 8 | |  | 14 | 0 |  | 29 | 1 |  | 33 | 0 |
|  | **Corynebacteriaceae** | |  |  |  |  |  |  | |  |  |  |  |  |  |  |  |  |
|  | | *Corynebacterium* sp.  (FJ374772) | 97.83 |  | 0 | 0 |  | 0 | |  | 0 | 0 |  | 0 | 0 |  | 2 | 1 |
|  | **Micrococcaceae** | |  |  |  |  |  |  | |  |  |  |  |  |  |  |  |  |
|  | *Kocuria* sp.  ( EU196302) | | 99.25 |  | 0 | 0 |  | 0 | |  | 0 | 0 |  | 0 | 0 |  | 2 | 0 |
| **Bacteroidetes** | | |  |  |  |  |  |  | |  |  |  |  |  |  |  |  |  |
|  | **Bacteroidia** | |  |  |  |  |  |  | |  |  |  |  |  |  |  |  |  |
|  | **Prevotellaceae** | |  |  |  |  |  |  | |  |  |  |  |  |  |  |  |  |
|  | *Prevotella* sp.  ( AY957555) | | 98.41 |  | 0 | 0 |  | 0 | |  | 0 | 0 |  | 1 | 0 |  | 0 | 0 |
| **Firmicutes** | | |  |  |  |  |  |  | |  |  |  |  |  |  |  |  |  |
|  | **Bacilli** | |  |  |  |  |  |  | |  |  |  |  |  |  |  |  |  |
|  | **Bacillaceae** | |  |  |  |  |  |  | |  |  |  |  |  |  |  |  |  |
|  | | *Bacillus* sp.  ( FJ755949) | 100 |  | 0 | 0 |  | 0 | |  | 0 | 0 |  | 2 | 0 |  | 0 | 0 |
|  | **Carnobacteriaceae** | |  |  |  |  |  |  | |  |  |  |  |  |  |  |  |  |
|  | | *Carnobacterium* sp.  (EU304249) | 97.86 |  | 0 | 0 |  | 0 | |  | 0 | 0 |  | 0 | 154 |  | 0 | 0 |
|  | **Streptococcaceae** | |  |  |  |  |  |  | |  |  |  |  |  |  |  |  |  |
|  | | *Streptococcus* sp.  ( AY518677) | 98.75 |  | 0 | 0 |  | 1 | |  | 3 | 0 |  | 3 | 0 |  | 0 | 0 |
|  | | *Lactococcus* sp.  ( AB118037) | 98.42 |  | 0 | 0 |  | 0 | |  | 0 | 0 |  | 0 | 2 |  | 0 | 0 |
|  | **Clostridia** | |  |  |  |  |  |  | |  |  |  |  |  |  |  |  |  |
|  | **Peptostreptococcaceae** | |  |  |  |  |  |  | |  |  |  |  |  |  |  |  |  |
|  | | *Anaerococcus* sp.  (AM176540) | 92.14 |  | 0 | 0 |  | 0 | |  | 0 | 0 |  | 0 | 0 |  | 4 | 0 |
